# Elucidating the Effects of Interconnecting Layer Thickness and Bandgap Variations on the Performance of Monolithic Perovskite/Silicon Tandem Solar Cell by wxAMPS

**DOI:** 10.3390/ma16114106

**Published:** 2023-05-31

**Authors:** Ili Salwani Mohamad, Camellia Doroody, Wabel Mohammed Alkharasani, Mohd Natashah Norizan, Puvaneswaran Chelvanathan, Seyed Ahmad Shahahmadi, Nowshad Amin

**Affiliations:** 1 College of Engineering, Universiti Tenaga Nasional, Jalan IKRAM-UNITEN, Kajang 43000, Malaysia; 2 Faculty of Electronic Engineering & Technology, Universiti Malaysia Perlis (UniMAP), Arau 02600, Malaysia; 3 Institute of Sustainable Energy, Universiti Tenaga Nasional, Jalan IKRAM-UNITEN, Kajang 43000, Malaysia; 4 Geopolymer and Green Technology, Centre of Excellent (CEGeoGTech), Universiti Malaysia Perlis (UniMAP), Arau 02600, Malaysia; 5 Solar Energy Research Institute, Universiti Kebangsaan Malaysia, Bangi 43600, Malaysia; 6 Engineered Nanosystems Group, School of Science, Aalto University, Tietotie 1, 02150 Espoo, Finland

**Keywords:** energy, solar cell, tandem, silicon, perovskite, wxAMPS, numerical simulation, monolithic, interconnecting layer

## Abstract

In this study, we investigated the pathways for integration of perovskite and silicon solar cells through variation of the properties of the interconnecting layer (ICL). The user-friendly computer simulation software wxAMPS was used to conduct the investigation. The simulation started with numerical inspection of the individual single junction sub-cell, and this was followed by performing an electrical and optical evaluation of monolithic 2T tandem PSC/Si, with variation of the thickness and bandgap of the interconnecting layer. The electrical performance of the monolithic crystalline silicon and CH_3_NH_3_PbI_3_ perovskite tandem configuration was observed to be the best with the insertion of a 50 nm thick (E_g_ ≥ 2.25 eV) interconnecting layer, which directly contributed to the optimum optical absorption coverage. These design parameters improved the optical absorption and current matching, while also enhancing the electrical performance of the tandem solar cell, which benefited the photovoltaic aspects through lowering the parasitic loss.

## 1. Introduction

Tandem solar cell technology has gained substantial interest in the research arena recently. Researchers have investigated different materials and structures to provide current matching for the maximum optical and electrical output, by improving the thermalization loss from single junction solar cells. Crystalline silicon is known to be the most stable solar cell and dominates the photovoltaic market. On the other hand, perovskite solar cells have demonstrated a convincing performance, moving toward the Shockley–Queisser (SQ) limit efficiency for single junction solar cells just a decade after they were first developed. The bandgap difference between these two cells makes them a potential candidate for a tandem configuration. However, when two p-n junctions are combined, the phenomenon of a counter electrode exists and consequently limits the current flow.

The most efficient and stable crystalline silicon (c-Si) solar cell, with a maximum power conversion efficiency (PCE) of 26.7%, is getting close to the theoretical maximum efficiency of 29.4%, and 4% below the Shockley–Queisser limit compared to the limit of other single junction cells, due to Auger recombination [1]. Dominating 95% of the photovoltaic (PV) market [2], c-Si as a single junction solar cell has moved closer to its detailed-balance theoretical limit. At the same time, there has been strong interest in using several semiconductor layers with different bandgaps in tandem configurations, to mitigate carrier thermalization losses and as an ideal approach to surpass c-Si solar cell’s SQ limit.

Metal halide perovskite solar cells (PSC) have shown rapid growth, with the current record efficiency of 25.2% being reported in 2019, increasing from the 3.8% when they were first developed in 2009 [3]. This stellar achievement of PSC technology is mainly attributed to its tunable band gap (1.5 to 2.3 eV), high carrier mobility, long carrier diffusion length, strong optical absorption, and high defect tolerance [4]. As the theoretical SQ efficiency limit for PSC is reported to be 33.7%, a perovskite solar cell is an ideal candidate to be integrated with c-Si technology for a tandem design. Although silicon heterojunction (SHJ) technology now dominates tandem research, homojunction c-Si solar cells are expected to perform better than SHJ PV devices [5], given the modest conduction band offset (CBO) and valence band offset (VBO) caused by the minor discrepancy of electron affinities in homojunction c-Si solar cells.

A tandem configuration device can be developed as a two-terminal (2T) (monolithic) or four-terminal (4T) (mechanically stacked) device. Two cells are optically and electrically connected in a 2T device. This connection reduces the expense of one or more transparent electrodes, by sharing the intermediate electrode. According to reports, a 2T tandem structure has stronger light coupling and less optical loss between the two sub-cells than a 4T device [6,7]. A 2T monolithic perovskite/silicon (PSC/Si) tandem device recorded high efficiency of 29.15% [8], compared to the 28.2% [9] in the 4T device. As reported by Ruhle, the detailed balance limits of 2T and 4T PSC/Si devices are 41.6% and 43.7%, respectively, considering a top band gap PSC cell of 1.55 eV [10]. The total current density (J_sc_) flowing in the tandem device is determined by the lowest short circuit current (J_sc_) output from any of the sub-cells, where the open circuit voltage (V_oc_) for tandem is the sum of the V_oc_ of the sub-cells.

**Figure 1:**
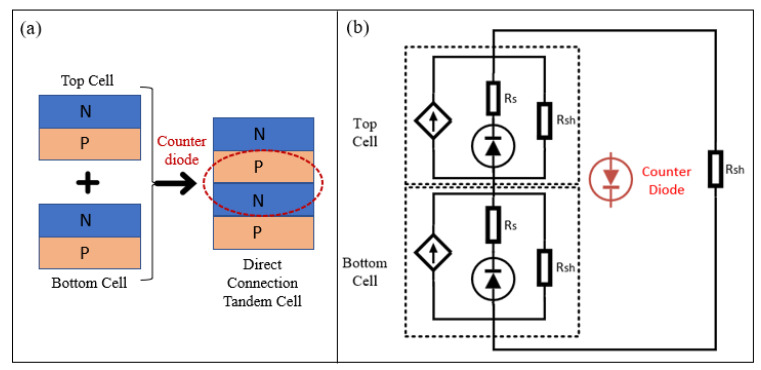
(**a**) Reverse diode formation, due to direct connection of two p-n junctions; (**b**) diode illustration of the reverse diode formation phenomenon in the equivalent circuit.

A counter p-n diode is formed when two p-n junctions are coupled, as shown in Figure 1, restricting the current flow. The barrier originating from the work function difference between the n-type material (usually having a lower work function for electron extractions) and the p-type material (usually having a higher work function for hole extractions) limits the current flow, even though the optical losses are reduced. In addition, it was also reported that the high recombination rate on an unpassivated emitter surface resulted in a significant electrical loss [11].

The first functional monolithic 2T tandem PSC/Si solar cell was developed by Mailoa et al. using an n-i-p structured CH_3_NH_3_PbI_3_-based PSC top cell and a homojunction crystalline Si lower cell with a n++ a-Si/p++ c-Si tunnel junction, resulting in an efficiency of 13.7% [12]. Mailoa claimed to have discovered the parasitic absorption loss, related to the reduction of J_sc_. Werner et al. reported in another work (2016) and described the usage of a zinc tin oxide (ZTO) interfacial layer but also observed series resistance, which led to fill factor reduction [13]. Thus, improved control of the charge carrier density in the interconnecting layer (ICL) is needed, to reduce the interfacial resistance. The ICL serves as a recombination layer that gathers carriers from the top and bottom sub-cells, while introducing an electric field directed in the opposite direction of the photovoltage and balancing the output voltage [6]. Thus, an ICL should possess a high optical transparency (especially in the 600 nm-visible to 1200 nm-near infrared region) [8], high electrical conductivity [14], high electron mobility [15], and a suitable refractive index for optimum light coupling into the bottom cell.

Tandem solar cells are an important breakthrough in photovoltaic technology, as they give potential for a PV device to surpass the Shockley–Queisser limit with a single junction cell. A good mechanical, optical, and electrical interconnection is demanded at the same time, to efficiently connect the two sub-cells in 2T tandem SCs. In a 2T tandem device, the current matching between the coupled cells is very important for an efficient device. Thus, the study of an optimized ICL layer is needed, to ensure that the parasitic absorption of long-wavelength photons due to free-carrier absorption in ICL (TCO) is avoided.

Numerical simulation has been proven to assist researchers, allowing a unique opportunity to optimize the material characteristics needed for an efficient photovoltaic device via tailoring materials/combinatorial analysis [16]. In this work, we performed numerical simulations on PSC and Si single junction cells, which served as a baseline, followed by the investigation of the electrical and optical influence of ICL thickness and bandgap variations on the performance of a monolithic 2T PSC/Si device. Field-dependent mobilities and sub-cell analysis are included in the latest version of wxAMPS 3 from 2018 [17]. Similar to wxAMPS, several solar cell simulation tools are available, including SCAPS, PC1D, and AFORS-HET. However, in addition to drift–diffusion models, only wxAMPS can incorporate a variety of tunneling principles, including trap-assisted, intra-band, and band-to-band mechanisms. Restrictions on the number of layers and defects were removed using dynamic memory allocation.

## 2. Modeling Procedure

**Figure 2:**
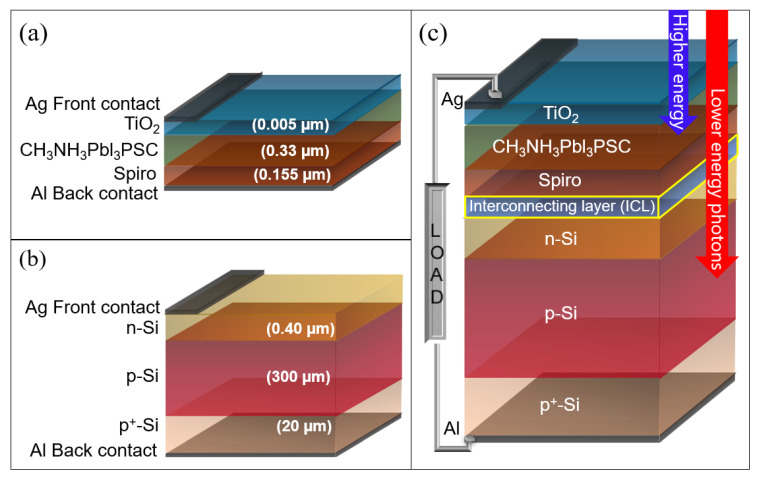
(**a**) perovskite solar cell, (**b**) silicon solar cell, and (**c**) tandem 2T PSC/Si solar cell.

Tandem solar cells, possessing PSC as the top cell, have attracted much attention, due to the rapid progress in power conversion efficiency. In this work, wxAMPS (analysis of microelectronic and photonic structures) software was adapted to model single and multi-junctions, homo- and hetero-junctions, as well as Schottky barrier devices, because it can solve dipolar problems using Poisson’s equation, the carrier continuity equation, and boundary conditions [18]. A theoretical investigation into the physical workings of a built-in photovoltaic structure under illumination or dark was made possible by numerical calculation. Figure 2 shows the baseline and tandem structures proposed in this work. The modeled devices were simulated under AM 1.5G solar spectrum illumination, striking the solar cells’ fore layers.

wxAMPS only considers non-degenerate semiconductors, and the carrier populations are subject to Boltzmann statistics. The trap-assisted tunneling (TAT) model implemented by wxAMPS is crucial for modeling tandem solar cells and other devices where the electric field is strong at highly doped junctions. Since the tunneling distance is relatively short at these junctions, there is a higher probability that electrons from the valence band tunnel through the defect or trap states and into the conduction band, increasing the current density. The TAT model alters the Shockley–Read–Hall (S–R–H) recombination form by incorporating tunneling functions. Here, tunnel junctions and negative resistance phenomena were accurately modeled using the band-to-band model, a recent addition to wxAMPS. This model, which is more accurate in describing multi-junction devices, is based on a non-local formulation of tunneling. For field-dependent carrier mobilities, the transferred electron model was adopted.

Furthermore, wxAMPS implements the absorption coefficient parameter, which encompasses the full solar spectrum. In quantum efficiency (QE) simulation, the transfer matrix method can be enabled if optical interference is present. The interference patterns are then be observable in the QE simulation output. Table 1 shows the cell characteristics used in this work, which were defined based on the theoretical principles, experimental data, and available literatures [19,20,21,22].

To ensure a smooth simulation process, the “sub-cell analysis (only for tandem)” box needs to be ticked (√) to avoid convergence failure. “Mesh Grid Edge” and “Center” set up under the “Advanced” tab for each layer need to be specified, based on the requirement outlined by the developer, where the x value in the following calculation needs to be satisfied.

(1) $$Grid\;Edge\;\to \;\frac{Total\;thickness\;\left(nm\right)\;}{x}=5000\;or\;less\;than\;8000\;$$

(2) $$Grid\;Centre\;\to \;\frac{Total\;thickness\;\left(nm\right)\;}{x}=250$$

Binary grids are often combined with a given number of meshes for spatial derivatives, and the meshes are defined for timed determinants [23]. Mesh density is one of the crucial factors in ensuring a simulation’s validity and accuracy. A well-defined grid is needed for tandem configuration simulation, which requires tunneling junction and current matching between the two sub-cells. Table 2 summarizes the grid values defined in this simulation. Equations (1) and (2) determine the grid edge and center when defining the mesh for each layer component of the tandem device.

**Figure 3:**
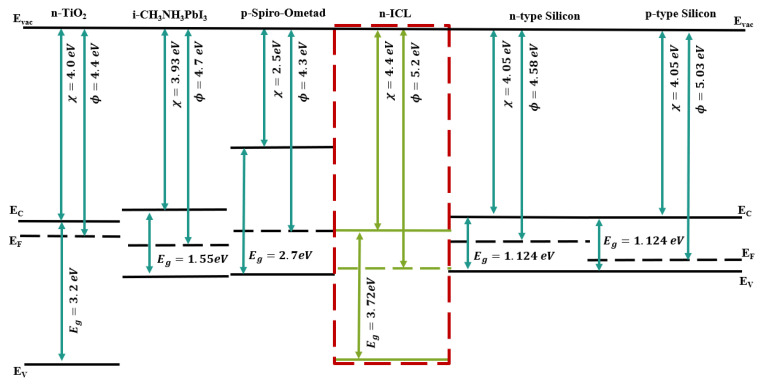
Band diagrams for all materials in the proposed PSC/Si 2T tandem solar cell.

A band diagram of the materials utilized in this work is shown in Figure 3, which included n-TiO_2_, intrinsic i-CH_3_NH_3_PbI_3_ perovskite, p-Spiro-Ometad, interconnecting layer (ICL), n-type and p-type silicon.

This study was divided into three stages: First, Si and PSC baseline cells were modeled, and the electrical parameters were compared with the proposed ICL integrated tandem design. Next, a modeling analysis was conducted, to illustrate the main changes in the electrical properties and efficiency of the tandem design with and without the ICL layer. By confirming the potential of using ICL, the final stage was to study the variations of ICL thicknesses and bandgaps. All other layer thicknesses and material properties were kept constant during each simulation. 

## 3. Results and Discussion

### 3.1. Single Junction and Tandem Cell Baseline Cell

**Figure 4:**
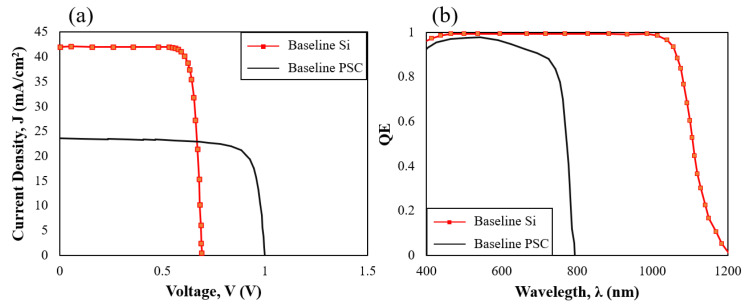
(**a**) J-V curve, and (**b**) QE comparison between the baseline Si and PSC devices.

An optoelectrical model was developed using wxAMPS to explain the characteristics of the individual Si solar cells and PSC. The current density (J_sc_) and quantum efficiency (QE) of each device using absorption data prior to tandemization are presented here, to evaluate potential improvements. Figure 4a indicates that the Si solar cell yielded a higher photo-current value at a lower voltage rate, while PSC generated a lower J_sc_ and higher V_oc_, owing to features such as a high carrier mobility and low trap density, which resulted in a reduced absorption rate at higher wavelengths. Figure 4b shows that PSC has optimal properties to be used as a top cell, attributed to its broader band energy absorbing short-wavelengths; as opposed to the Si cell, which is suitable for a bottom cell, absorbing long-wavelength photons and generating a photo-current at a lower voltage. QE plots for the silicon solar cell and perovskite cell agreed precisely with the equation λ = hc/E_g_; whereby, theoretically, perovskite with E_g_ = 1.55 eV and silicon with E_g_ = 1.124 eV absorb photons with wavelengths up to 800 nm and 1200 nm, respectively. Both QE patterns matched the trend reported by NREL for Si [24,25] and fabricated PSC cells [26].

Table 3 displays important parameters related to the benchmark silicon and CH_3_NH_3_PbI_3_-based perovskite solar cells, from reliable experimental literature reports, as well as the findings of our investigation. Here, the individual modeling output results showed that the baseline Si had a higher I_mp_ of 41.07 mA/cm^2^ and a maximum power point (MPP) of 24.35 W at 0.59 V; while the baseline of PSC revealed an I_mp_ of 21.20 mA/cm^2^ and MPP of 18.76 W at 0.88 V, indicating a weak dynamic of PSC, which might have caused the current limits in the tandem design, in-line with previous reports [27]. CH_3_NH_3_PbI_3_-based perovskite was chosen in this work, as it has been the main perovskite material used since single junction cells of this technology were developed, and sufficient literature describing the material properties was found, as a basis for this numerical simulation work. 

The results in Table 3 imply that, when simulating a solar device, the optical model can almost predict the ideal characteristics of the fabricated single cell device, considering the effect of defects [21,30]. However, optical and electrical factors also need to be considered, to explain the divergence from experimental data and acquire more insights into tandem device configuration and performance.

### 3.2. Tandem Cell Design with and without an Interconnecting Layer

**Figure 5:**
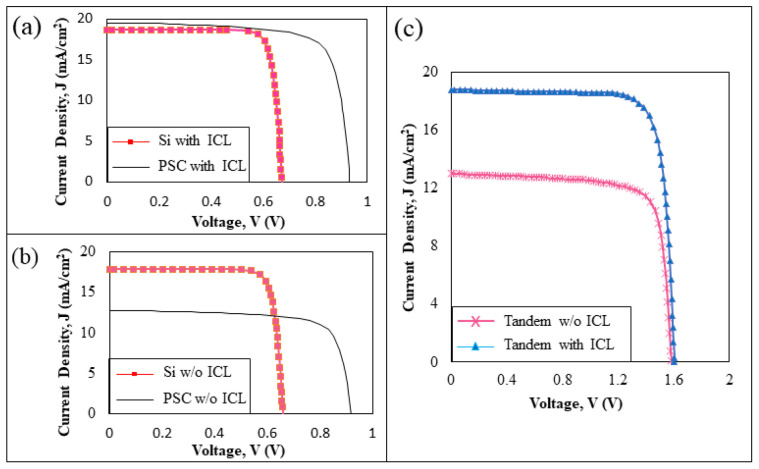
J-V curves for a silicon/perovskite tandem cell (**a**) with ICL, (**b**) without ICL, and (**c**) with and without ICL comparison.

Figure 5a,b compare the individual characteristics of Si and PSC, configured with and without ICL layers. Not much difference was found in terms of V_oc_ for both devices in both tandem configurations, but a J_sc_ reduction can be observed compared to its single junction cell performance. The tandem design of Si and PSC with no ICL resulted in 17.83 mA/cm^2^ and 12.74 mA/cm^2^ photocurrents, respectively. An improvement in J_sc_ performance for both the Si and PSC tandem design resulted from using the ICL layer, where the devices recorded 18.65 mA/cm^2^ and 19.51 mA/cm^2^, respectively. Clear effects of the ICL on the total J_sc_ performance are depicted in Figure 5c, where the tandem device with ICL recorded a current matching at 18.77 mA/cm^2^; however, only 13.01mA/cm^2^ was found for the non-ICL tandem device. The optimized V_oc_ for both tandem structures, with and without ICL, was maintained at 1.6 V. Based on these results, we can conclude that the total J_sc_ in a tandem device is equivalent to the lowest J_sc_ that flows through either one of the sub-cells, to ensure current matching; and V_oc_, on the other hand, is the sum of the two sub-cells.

The simulation-derived impacts of the ICL insertion parameters on the structure could be used as a guideline for the experimental procedure. The performance characteristics shown in Table 4 reveal a significant improvement in efficiency when utilizing the ICL, despite the V_oc_ value showing only a slight change. The optimized simulated tandem device performance with ICL matched the reported fabricated cell. This supports the authenticity of the findings and the overall scope of this work as the baseline cell for a silicon, perovskite, and tandem cell device, as well as the ICL parameter variation investigations.

### 3.3. Electrical Performance Effect of Bandgap and Thickness Variations on the PSC/Si Tandem Configuration

**Figure 6:**
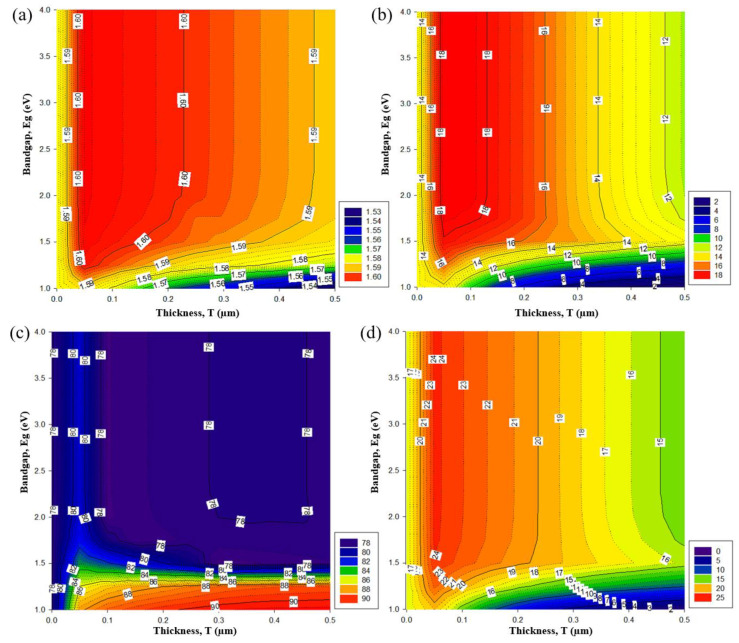
The photovoltaic parameters: (**a**) V_oc_, (**b**) J_sc_, (**c**) FF, and (**d**) efficiency of the tandem device with ICL as a function of E_g_ and T.

Subsequently, to minimize the parasitic absorption and recombination, the ideal bandgap and thickness of the possible candidates for the ICL were explored for the modeled two-terminal tandem structures. The electrical performance of the device is shown in Figure 6, while adjusting the thickness (T) and bandgap (E_g_) of the interconnecting layer, to acquire the optimized properties for the ICL material.

The thickness of the ICL cannot be zero because this induces recombination, which is detrimental to the carrier collection process [12]. With a thickness increment from 0 to 500 nm, the V_oc_ and J_sc_ rapidly increase to the saturation value at 50 nm. A thicker ICL induces recombination and adverse absorption. Even though the changes in V_oc_ with the thickness variations were small, as in Figure 6a, the J_sc_ improved from 1.1 to 18.8 mA/cm^2^ (Figure 6b), the fill factor improved from 77.08 to 90.55% (Figure 6c), and the efficiency improved by approximately 22.79%, from 1.54 to 24.32% (Figure 6d). A higher bandgap, E_g_ ≥ 2.5 eV, induced a short wavelength absorption loss; and a low bandgap, E_g_ ≤ 1.5 eV, induced thermalization loss, due to absorbing higher energy photons [32]. Overall, the higher recombination values using any materials with bandgaps below 1.5 eV and/or a thickness of ICL ≥100 nm decrease the V_oc_, J_sc_, and consequently the efficiency across the tandem device, which reduces the voltage and electric field across both sub-cells, lowering the charge collection ɳ, J_sc_, and FF. The results from this analysis were consistent with prior reports [33]. In summary, specifically for a c-Si/MAI PSC tandem configuration, the highest efficiency was obtained for the cell with an ICL thickness of 50 nm and E_g_ ≥1.75 eV. Optimum electrical performances of 1.61 V V_oc_, 18.52 ≤ J_sc_ ≤ 18.77 mA/cm^2^, 81% FF, and 24.32% efficiency were obtained. 

### 3.4. Optical Performance Effect of Bandgap and Thickness Variations on the PSC/Si Tandem Configuration

**Figure 7:**
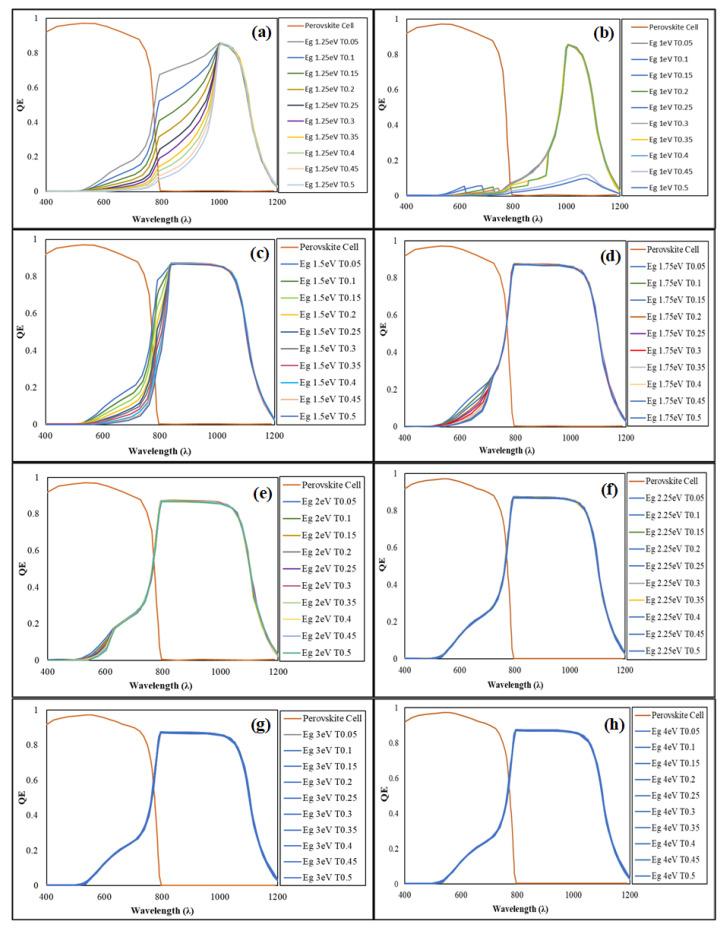
Tandem QE with ICL thickness variations and (**a**) E_g_ of 1 eV, (**b**) E_g_ of 1.25 eV, (**c**) E_g_ of 1.5 eV, (**d**) E_g_ of 1.75 eV, (**e**) E_g_ of 2 eV, (**f**) E_g_ of 2.25 eV, (**g**) E_g_ of 3 eV, and (**h**) E_g_ of 4 eV.

In a tandem configuration, the wide-bandgap material captures photons with higher energies, while the remaining photons with lower energies traverse the top cell and are absorbed by the rear sub-cell [34]. Figure 7 represents the ICL influence on the total absorption spectrum, based on the bandgap energy specified and the thickness of the proposed ICL material. The influence of ICL thickness variations is significant at 1 < E_g_ < 2 eV and is negligible for E_g_ ≥ 2.25 eV. An ICL with a low bandgap tends to absorb part of the spectrum, before the high wavelength photons are transmitted to the rear cell, as shown in the trend observed in Figure 7a–e. QE trends are constant for E_g_ ≥ 2.25 eV, where PSC and Si absorb lower and higher wavelength photons, respectively, reducing the thermalization loss. Here, a thickness of 50 nm, resulting from the analysis in Figure 7 with E_g_ ≥ 2.25 eV, can be specified as the optimum characteristic for any potential ICL material for use in a PSC/Si tandem setup.

**Figure 8:**
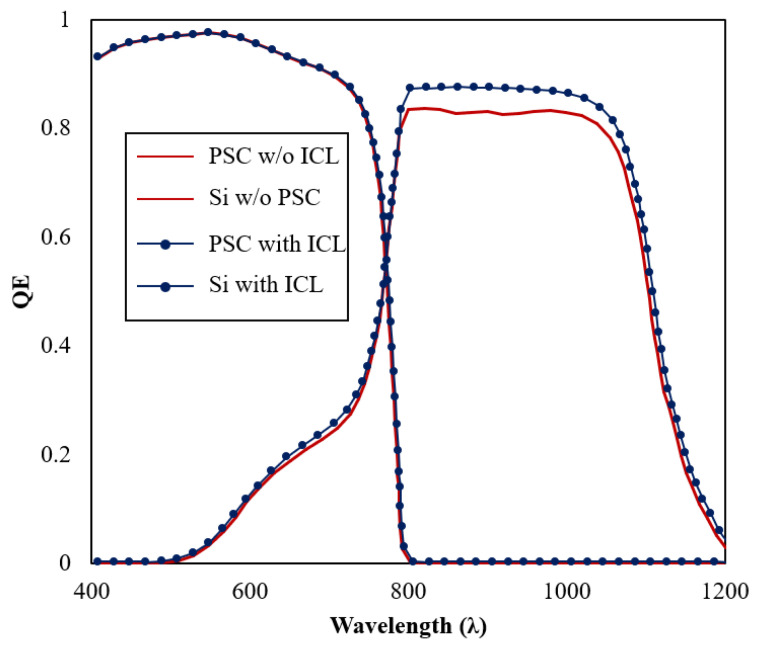
QE spectra of Si and PSC sub-cells of the proposed tandem configuration.

Computational results from tandem design simulations, with and without the addition of an ideal ICL layer (E_g_ ≥ 2.25 eV, T = 50 nm), are shown in Figure 8, to demonstrate the influence of ICL on the absorption and photon transport mechanism improvement.

A PSC cell absorbs high-energy blue and green light, while a Si cell function well in red and infrared light regions. The collective QE for the top cell (PSC) at shorter wavelengths, up to 700 nm, surpasses 95%. Meanwhile, with an ICL, the bottom cell or Si device exhibits a substantial reduction of the reflection loss and an improved diffusion length, resulting in an overall efficiency improvement of the cells. As the PSC is the first cell to receive photons, no difference in the QE trend is observed. Absorption reduction in Si cells was detected in tandem, without an ICL, compared to with an ICL. This absorption reduction impacted the J_sc_ performance of the tandem device. Here, we can see that a tandem design minimizes the thermalization loss in silicon devices, as the high-energy electrons at the lower wavelength of the light spectrum can be utilized by the higher bandgap material, to improve electron generation. Apart from using PSC, porphyrin materials can also be considered as one of the top cell candidates for a tandem design, owing to its remarkable features of a good thermal stability, efficient electron transport mechanism, air stability, optical and electronic properties that can easily be tuned using straightforward synthetic modifications, and being an excellent light harvester [35].

## 4. Conclusions

Optimal interconnecting layer (ICL) material properties are indispensable in realizing high-performance monolithic perovskite/silicon tandem solar cells. In this work, numerical simulation on the impact of an ICL, particularly ITO as a function of various thicknesses (T) and energy bandgaps (E_g_), was explored via the wxAMPS tool. Besides tandem design configurations with and without an ICL layer, single-junction silicon and perovskite solar cells have also been constructed and evaluated. The best efficiency of 24.32% was observed in the PSC/Si tandem design with an ICL layer, which was 51.81% higher than the simulated tandem device without an ICL and 14.72% higher compared to the fabricated tandem device. This high-performance device was derived from the optimum regions, with the E_g_ and T for the ICL were ≥2.25 eV and 50 nm, respectively. In contrast, the QE trends showed that thicker and lower bandgap ICL materials tend to absorb some photons rather than fully transmitting them to the bottom cell, resulting in a reduced electrical performance. Finally, these findings were successfully correlated with the thermalization loss reduction, by utilizing different material bandgaps in a tandem configuration in a PV device.

## Figures and Tables

**Table 1:** Physical and electronic specification of the materials/layers used in the simulation [19,20,21,22].

Material Properties	n-TiO_2_	i-MAI PSC	p-Spiro Ometad	ITO (ICL)	n-Si	p-Si	p^+^-Si
Thickness (µm)	0.005	0.330	0.155	**0–0.05**	0.400	300	20.000
Dielectric permittivity ε/ε_0_	9.0	6.5	3.0	9.4	11.9	11.9	11.9
Bandgap E_g_ (eV)	3.200	1.550	2.700	**1.00–4.00**	1.124	1.124	1.124
Electron affinity χ (eV)	4.00	3.93	2.50	3.60	4.05	4.05	4.05
C_B_ density of state (×10^19^) (cm^−3^)	67.70	0.28	1.00	0.22	2.85	2.85	2.85
V_B_ density of state (×10^19^) (cm^−3^)	13.00	0.39	1.00	1.80	1.04	1.04	1.04
Electron mobility (cm^2^ V^−1^s^−1^)	0.02	12.50	0.0002	100	1350	1350	1350
Hole mobility (cm^2^ V^−1^s^−1^)	0.02	7.50	0.0002	25	450	450	450
N_D_ density (cm^−3^)	1 × 10^20^	5.21 × 10^9^	0	1 × 10^19^	2.5 × 10^19^	0	0
N_A_ density (cm^−3^)	0	5.21 × 10^9^	1 × 10^20^	0	0	1 × 10^16^	1 × 10^18^
Defect (band tails) E (conduction, valence) eV	na	0.045, 0.045	na	na	na	na	na
G_o_ (conduction, valence) 1/cm^2^/eV	na	1 × 10^19^, 1 × 10^19^	na	na	na	na	na
σ_N_ (conduction, valence) cm^2^	na	1 × 10^−13^, 1 × 10^−14^	na	na	na	na	na
σ_P_ (conduction, valence) cm^2^	na	1 × 10^−14^, 1 × 10^−13^	na	na	na	na	na

**Table 2:** Mesh grid edge and center specification of layers used in the simulation.

Material Properties	n-TiO_2_	i-MAI PSC	p-Spiro Ometad	ITO (ICL)	n-Si	p-Si	p^+^-Si
Grid Edge	0.001	0.066	0.031	varied	0.1	50	4
Grid Center	0.02	1.32	0.62	varied	2	1500	80
Band to Band Recombination	2.31 × 10^9^	0	2.31 × 10^9^	0	0	0	0
Tunneling Effective Mass (m-c and m-v)	1	1	1	1	1	1	1

**Table 3:** Photovoltaic modeling and experimental outputs for baseline Si and PSC.

Cell Type	V_oc_ (V)	J_sc_ (mA/cm^2^)	FF (%)	ŋ (%)	Ref
C-Si single cell	0.69	42.08	84.38	24.53	this work
UNSW, p-type PERC	0.71	42.70	82.80	25.0 +/− 0.5	[28]
TiO2/MAPbI_3_/Spiro-Ometad	1.03	23.56	79.48	19.27	this work
ITO/Li, Ag:NiO_x_/MAPbI_3_/PCBM/Ag	1.13	21.29	80.00	19.24	[29]

**Table 4:** Simulated device efficiency, and J-V parameter comparison with the literature.

Cell Type	V_oc_ (V)	J_sc_ (mA/cm^2^)	FF (%)	ŋ (%)	Ref
Tandem PSC/c-Si no ICL	1.58	13.01	77.89	16.02	this work
Tandem PSC/c-Si with ICL	1.61	18.77	80.64	24.32	this work
UNIST/KIST PSC/Al-BSF with ICL	1.65	16.10	79.90	21.20	[31]

## Data Availability

Not applicable.
